# Effectiveness of an immersive virtual reality intervention to reduce anxiety during pregnancy: a quasiexperimental single-group pre-post study

**DOI:** 10.1186/s12884-025-08097-8

**Published:** 2025-09-24

**Authors:** María Caballero-Galilea, Laura Esteban-Gonzalo, Sara Esteban-Gonzalo, Juan Luis González-Pascual

**Affiliations:** 1 Faculty of Medicine, Health and Sports, Department of Nursing, Universidad Europea de Madrid, c/ Tajo s/n, Villaviciosa de Odón, Madrid, 28670 Spain; 2 Faculty of Nursing, Physiotherapy and Podiatry, Department of Nursing, Universidad Complutense de Madrid, Av. Complutense s/n, Madrid, 28040 Spain; 3 Faculty of Psychology, Department of Social, Work and Differential Psychology, Universidad Complutense de Madrid, Ctra. de Húmera, s/n, Pozuelo de Alarcón, Madrid, 28223 Spain

**Keywords:** Pregnant people, Virtual reality exposure therapy, Anxiety, Anxiety disorders, Pregnancy-related anxiety, Prenatal education

## Abstract

**Background:**

Pregnancy-related anxiety affects a significant number of women and has adverse consequences for both mothers and children. Virtual reality has emerged as an innovative strategy in the field of mental health to address anxiety disorders. However, in the field of obstetric healthcare, it has mostly been used as a distraction tool. The aim of this research was to determine the effectiveness of an immersive virtual reality intervention in reducing pregnancy-related anxiety during the third trimester of pregnancy.

**Methods:**

A single-group pre-post quasiexperimental study was conducted with 73 pregnant women who participated in a prenatal education program at a health center in Madrid. The intervention consisted of an interactive and immersive simulation with virtual reality, which was carried out during the last trimester of pregnancy. Anxiety was measured before and after the intervention via the Pregnancy Related Anxiety Questionnaire (PRAQ-20). The data were analyzed using descriptive and inferential statistical tests, including logistic regression.

**Results:**

The median age of the participants was 34 years. Most were university educated and had no previous children. The results revealed a statistically significant decrease (*p* < 0.001) in the pregnancy-related anxiety score following the intervention. According to the logistic regression model, the intervention significantly increased the probability of reducing anxiety (*p* < 0.047), adjusting for other variables.

**Conclusions:**

Compared with other interventions that have demonstrated efficacy in decreasing pregnancy-related anxiety, such as cognitive behavioral therapy, mindfulness, and others, immersive VR intervention offers the advantage of being a brief and unique intervention. In conclusion, it could be an effective tool for reducing pregnancy-related anxiety.

## Background

Anxiety during pregnancy is a problem that affects a significant number of women worldwide, ranging from 18 to 25% on average depending on the trimester of pregnancy if we consider anxiety symptoms and 15% on average if we refer to a clinical diagnosis of anxiety disorder [1]. Its consequences affect both obstetric outcomes and the health of mothers and children. It is associated with increased induction of labor and increased use of drugs during labor [2], a higher frequency of admission to neonatal intensive care, worse Apgar scores, increased hospital stay [3], a higher rate of preterm births, low birth weight [4, 5], an increased risk of postpartum depression, a lower rate of breastfeeding [6] and an increased likelihood of developing psychopathology in childhood [7].

It is related to multiple factors, including obstetric, psychological and social factors, such as preoccupied maternal attachment [8], complications in previous pregnancies, a desire to become pregnant, a history of miscarriage or threatened preterm birth [9], unplanned pregnancy, a lack of social support [10], and a lack of emotional stability and introversion [11].

One of its dimensions is fear of childbirth, which affects 5–15% of pregnant women [12, 13]. It is a problem defined by three critical characteristics: cognitive impairments, affective disorders and somatic symptoms [14]. It is related to different personal and social aspects, such as fear of the unknown, uncertainty of how the birth will go, fear of loss of control, and fear of complications, such as pain or injury [15], lack of social support, and negative experiences in previous pregnancies or births [16]. In addition to other problems, this may be related to an increase in the request for and performance of cesarean Sect. [16].

Structured birth preparedness programmes, although they vary widely in the target population, professionals involved, and interventions across the world are effective in improving maternal/infant outcomes and behavioral or lifestyle issues [17]. In addition to interventions aimed at physical health, such as supplements, vaccinations, screening, information on pregnancy and childbirth, and assessment of fetal development [18], interventions aimed at the well-being and mental health of pregnant women, such as mindfulness [19, 20], breathing techniques, yoga and cognitive‒behavioral therapy [21], can be included. In this sense, pregnant women demand not only maintaining a healthy pregnancy for mothers and babies but also an effective transition to positive labor and birth and achieving positive motherhood (including maternal self-esteem, competence, autonomy) [22].

In recent years, virtual reality has emerged as an innovative strategy in the mental health field to address anxiety disorders. Desensitization through controlled exposure in VR environments facilitates progressive adaptation to stressful situations [23].

In the field of obstetric health, virtual reality has been used for several purposes [24], such as facilitating physical exercise during pregnancy [25], reducing anxiety during first trimester surgical abortion [26], reducing pain in episiotomy repair [27], reduce anxiety during nonstress tests [28], and especially as a distraction to reduce pain and/or anxiety at the time of giving birth - both vaginal and cesarean delivery – [29–32]. In the context of mental health, virtual reality interventions have primarily aimed to reduce anxiety in hospital settings, either during medical procedures or at the time of childbirth. A recent systematic review confirmed the positive impact of these interventions in reducing anxiety among pregnant women in such environments [33].

However, research on the use of virtual reality to improve mental health during pregnancy—outside of procedural or childbirth settings—and specifically to reduce pregnancy-related anxiety and fear of childbirth, remains limited.

In relation to anxiety in pregnant women—excluding pregnancy-specific anxiety—recent studies have demonstrated the effectiveness of virtual reality interventions that simulate natural environments [34] or green spaces [35].

Regarding pregnancy-related anxiety in specific populations, virtual reality visualization of the fetus has been shown to reduce anxiety in women with unwanted pregnancies [36].

More broadly, in efforts to promote mental health among pregnant women, a limited number of studies suggest that virtual reality may be effective in reducing anxiety. These include fetal visualization [37], a six-week mindfulness program delivered via virtual reality in 14-minute sessions [38], and a psychological activity program consisting of five sessions per week over five weeks [39].

These studies are few and very recent, with most not yet published at the time this research was initiated. Moreover, they do not appear to specifically address pregnancy-related anxiety or fear of childbirth, highlighting the need for further investigation in this area.

## Methods

### Aim

The aim of this research is to determine whether desensitization through if controlled exposure to the birth process via an immersive experience that includes virtual reality as an intervention is effective in reducing anxiety related to pregnancy in third-trimester pregnant women.

#### Design

Single-group pre-post quasiexperimental, study. Transparent Reporting of Evaluations with Nonrandomized Designs (TREND) guidelines were followed.

#### Study population

Pregnant women attending a prenatal education programme in a health center in Madrid (Spain) between February 2023 and October 2024 who participated in the immersive virtual reality intervention.

The prenatal education programme starts at week 26 and runs for a period of three - four weeks, with a total of eight hours of training. It consists of information about the childbirth process, from the earliest signs of labor to the expulsive phase, breathing and relaxation techniques, methods for pain relief, including both pharmacological and non-pharmacological approaches, advice and techniques to support breastfeeding, and information regarding maternal recovery following childbirth, newborn care, and the role of parents during the postpartum period.

Inclusion Criteria: Aged 18 years or older, non-multiple pregnancy, be either primiparous or multiparous in the third trimester of gestation.

Exclusion Criteria: Women with visual or hearing impairments that prevent effective participation in virtual reality experiences, or those with neurological, otorhinolaryngological, or mental health conditions that could be exacerbated using virtual reality devices.

#### Sample size calculation

Considering an intermediate effect size (d = 0.6), a significance level of 0.05 and a statistical power of 90% (1-β), a minimum of 69 women was calculated to detect significant differences in anxiety during pregnancy.

#### Sample and sampling

Seventy-three women were selected via nonprobabilistic, consecutive sampling.

### Description of the intervention

The intervention takes place after the completion of the previously described prenatal education programme in the last trimester of pregnancy.

It consists of an interactive and immersive simulation that includes virtual reality using Meta Quest 3 glasses, featuring a specially designed program developed by expert midwives and used with permission.

It stimulates not only the sense of sight and hearing through virtual reality but also kinesthetically through the accompaniment of a specialized assistant (midwife) and the use of various materials (fitball, fetal heart rate sensor, monitor tape, baby replica, peripheral venous line, compressor, wet gauze, syringe) and furniture (stretcher, birthing chair). During the simulation, pregnant women experience different situations of euthocic birth in a safe and controlled environment, including initial contractions, expulsion of the mucus plug, rupture of the amniotic sac, arrival at the hospital with the reception of the midwife for the birth process, passage to the birth room with fetal monitoring, cannulation of the peripheral venous line, review of giving birth positions, management of contractions with breathing, administration of epidural anesthesia, and the birth process by means of pushing and expulsion of the baby.

The intervention took place in a soundproofed room of approximately 430.56 square feet (40 square meters) within the health center, maintained at a temperature of around 73.4 degrees Fahrenheit (23 degrees Celsius), with soft indirect lighting, and ensuring the participants’ privacy.

It lasts 60 min, with 10 min of prebriefing, 40 min of simulation and another 10 min of debriefing at the end. The intervention focused on simulating the childbirth experience as previously described, without including structured psychological education related to anxiety.

### Variables

Dependent variable: Pregnancy-related anxiety before and after the intervention.

The independent variables used were previous pregnancies and previous abortions (including miscarriages and abortions).

Covariates (sociodemographic variables): Age and educational level (primary – elementary school, secondary – high school, or higher education - university).

### Data collection instruments

To measure pregnancy-related anxiety, the validated brief Spanish version of the Pregnancy-Related Anxiety Questionnaire, PRAQ-20 [40], was used. It consists of 20 questions with 5 response options on a Likert scale (ranging from 20 to 100 points). The higher the score is, the greater the degree of anxiety. It has a 5-factor structure: worry about changes in oneself (physical), fear of the baby’s integrity, feelings about oneself, fear of childbirth and worries about the future. Its reliability is 0.91 in the first trimester of pregnancy and 0.92 in the second and third trimesters. It has the same factor structure and reliability in nulliparous and multiparous women [40]. It was measured immediately before and after the intervention via an online form.

The remaining variables, i.e., age (completed years), previous pregnancies (yes/no), previous miscarriages/abortions (yes/no) and educational level (university, secondary, primary), were self-reported collected via an online form before the intervention.

### Statistical analysis

First, the fit of the quantitative variables to a normal distribution was calculated via the Kolmogorov‒Smirnov test. Following the results, descriptive statistics were performed for all variables, using medians and interquartile ranges for quantitative variables and absolute and relative frequencies (percentages) for qualitative variables.

Bivariate inferential statistics were subsequently performed via the Wilcoxon signed-rank test for related samples to assess the variation between preintervention and postintervention pregnancy-related anxiety (PRAQ-20) scores. Bilateral differences were considered significant at *p* < 0.05. The effect size of the Wilcoxon test was calculated.

Finally, multivariate analysis was performed via binary logistic regression. For this purpose, the variable “decrease in pregnancy-related anxiety” (yes/no) was defined on the basis of the difference in each pregnant woman’s PRAQ20 scores pre- and postintervention. Several adjusted models were calculated. For this purpose, the qualitative polytomous variable “educational level” was transformed into a dummy variable. All variables: age, educational level (dummy variable: 1 = university education, 0 = no university education), used as a proxy for educational level, previous pregnancies, previous miscarriages/abortions were initially included and removed one by one, and the level of significance and changes when each variable was removed were recorded. The regression model was estimated using the Backward: Conditional method. This stepwise approach begins with all covariates included and iteratively removes the least significant variable based on the Likelihood Ratio (LR) test, provided its probability of removal exceeds the threshold of 0.20. The maximum number of iterations allowed for model convergence was 20, ensuring sufficient opportunity for the algorithm to reach a stable solution. None of the variables were statistically significant at any of the steps, and the significance of the remaining variables was not altered by dropping them. Only the intervention (constant) was significant at all steps. The proportion of variance explained by the model was assessed via Nagelkerke’s R^2^. Goodness-of-fit was calculated via the Hosmer–Lemeshow test. Finally, the adjusted model with all the variables is presented because it explains the greatest variance and presents the highest goodness-of-fit, and because controlling for such variables ensures that the observed associations are not distorted by underlying confounding influences.

IBM SPSS v29 statistical software was used.

### Ethical aspects

A favorable report was obtained from the Clinical Research Ethics Committee. An information sheet was provided, and written informed consent was requested when all participants were invited to take part in the research. Additionally, in the online form, consent was again requested to proceed with the data collection.

## Results

Descriptive data for the sample are presented in Table 1. The median age was 34 years (range 21–49 years). Most had a university education and no previous children.

**Table 1:** Characteristics of the participants

*n*	73
Age ^a^	34.0 (IQR 5.0)
Educational level ^b^ University High school Elementary school	78.0% 20.6% 1.4%
Previous pregnancies ^b^ Yes No Previous miscarriages/abortions ^b^ Yes No	24.7% 75.3% 26% 74%

^a^ Values are median (interquartile range). ^b^ Values are percentage

The results of the PRAQ20 questionnaire before and after the intervention are presented in Table 2. There was a statistically significant decrease (*p* < 0.001) in the pregnancy-related anxiety score. The effect size was large (> 0.5). For 58 women, the score decreased following the intervention; for 4, there was a tie; and for the remaining 11, the score increased.

**Table 2:** Pregnancy-related anxiety (PRAQ20) before and after the intervention

	Before intervention	After intervention	*p* value^a^	Effect size
PRAQ20 score^a^	49.0 (IQR 16.0)^b^	43.0 (IQR 12.5)^b^	**< 0.001**	0.69

^a^ Wilcoxon signed rank test for related samples. ^b^ Median (interquartile range)

Statistically significant values (p<0.05) are in bold

The logistic regression model is presented in Table 3.

After receiving the intervention, women were significantly (*p* < 0.047) more likely to have decreased levels of pregnancy-related anxiety, after adjusting for age, educational level, previous pregnancies and previous miscarriages. Nagelkerke’s R^2^ was 0.11. The Hosmer and Lemeshow test indicated a significance level of 0.75.

**Table 3:** Logistic mix model

Decrease in pregnancy-related anxiety		Adjusted model			
	OR		95% CI	*P*	
Intervention	237.6			**0.047**	
Age	0.93		0.81–1.07	0.292	
Educational level university	0.18		0.02–1.56	0.120	
Previous pregnancies Previous miscarriages/abortions	0.60 1.27		0.15–2.38 0.28–5.78	0.468 0.759	

Statistically significant values (*p* < 0.05) are in bold. Adjusted Model: Analyses were adjusted for age, educational level, previous pregnancies and previous miscarriages/abortions

## Discussion

The aim of this research was to determine whether the use of an immersive experience that includes virtual reality is effective in reducing pregnancy-related anxiety in pregnant women. The results show that the intervention decreases pregnancy-related anxiety scores, as measured by the PRAQ20 test, in pregnant women. The effect size was high in both bivariate and multivariate analysis, but the explanatory power was low in the multivariate analysis, indicating that there are more factors involved that have not been considered.

These results are consistent with the reduction in anxiety demonstrated by other interventions involving virtual reality, albeit linked to the timing of different procedures, such as bronchoscopy [41], episiotomy repair [42], nonstress tests [28], and childbirth [22, 29–31].

They are also consistent with the reduction of anxiety in pregnant women through virtual reality that simulate natural environments [34] or green spaces [35]. However, these interventions do not specifically target pregnancy-related anxiety or fear of childbirth.

Compared with the limited number of virtual reality interventions aimed at promoting mental health during pregnancy, the results of this study align with those obtained through a mindfulness program [38] and a psychological activity program [39]. Nevertheless, those interventions assessed anxiety reduction using general measures such as the State-Trait Anxiety Inventory (STAI) [38] and the Generalized Anxiety Disorder-7 (GAD-7) [39], which evaluate overall anxiety levels. In contrast, the Pregnancy-Related Anxiety Questionnaire (PRAQ-20) used in this research specifically measures anxiety associated with pregnancy, including fear of childbirth as one of its dimensions. Furthermore, the nature of the intervention differs. While mindfulness and psychological activity programs aim to promote general mental health and reduce anxiety—even within the specific population of pregnant women—the intervention in this research involves controlled exposure to the birth process, functioning as a form of desensitization. It is specifically designed to reduce pregnancy-related anxiety and fear of childbirth.

A similar distinction applies to interventions involving virtual reality interaction with a fetal model, both in pregnant women [37] and in those experiencing unintended pregnancies [36]. These interventions also showed efficacy in reducing anxiety, but the underlying mechanism was different from that in this research, as it did not involve desensitization through controlled exposure to the process of birth but rather distraction through visual and kinesthetic interactions with a 3D fetal model.

On the other hand, other interventions not involving virtual reality have also been used to reduce anxiety or fear of childbirth. Some of them have shown efficacy, such as attending childbirth preparation classes [43] and cognitive behavioral therapy, both alone [44] and in combination with physical exercise [45] and mindfulness [46]. A systematic review [47] reported that, of the interventions analyzed, cognitive behavioral therapy, mindfulness, biofeedback and peer mentoring were effective in reducing anxiety during pregnancy.

Compared with these other interventions - and despite the limitations in internal and external validity discussed in the limitations section, as well as the recognition that factors beyond the intervention itself may influence anxiety reduction - this immersive virtual reality intervention appears promising in reducing pregnancy-related anxiety. Moreover, it also has the advantage of requiring less time commitment for pregnant women, as it is a single brief intervention, whereas other interventions generally require multiple sessions over time.

### Limitations and future lines of research

This study is not without limitations. The measurement of pregnancy-related anxiety, although conducted via a validated questionnaire, is self-referenced. The absence of a control group increases the possibility of bias since it does not allow confirmation that the change in anxiety score is due to the intervention or to other uncontrolled factors, such as the placebo effect; however, the collection of data immediately before and after the intervention (1 h difference) limits the influence of other factors (recall bias) on the variation in the pregnancy-related anxiety score. Furthermore, the fact that the sampling was non-probabilistic and that the sample was composed predominantly of university-educated women limits the generalizability of the results.

Future research should evaluate whether the effects of the intervention are sustained in the medium term, particularly at the time of birth, and investigate its potential impact on birth outcomes (e.g., cesarean delivery, low birth weight). Additionally, its effectiveness should be examined across diverse sociodemographic groups, including women with lower educational attainment.

## Conclusion

This study offers preliminary evidence that a single-session immersive virtual reality intervention, designed to simulate the childbirth experience through controlled exposure, may be effective in reducing pregnancy-related anxiety during the third trimester. While the findings are promising, they should be interpreted with caution due to limitations in internal and external validity, including the absence of a control group and the non-probabilistic sampling of predominantly university-educated participants. Unlike other interventions that require multiple sessions and focus on general anxiety or mental well-being, this approach specifically targets pregnancy-related anxiety and fear of childbirth. Further research is needed to confirm these results, assess long-term effects, and explore its applicability across more diverse populations.

## Data Availability

The datasets generated and analyzed during the current study are available in the EU Open Research Repository (Zenodo), doi:10.5281/zenodo.15599829.
